# Serum Circ‐FAF1/Circ‐ELP3: A novel potential biomarker for breast cancer diagnosis

**DOI:** 10.1002/jcla.24008

**Published:** 2021-09-21

**Authors:** Ramtin Omid‐Shafaat, Hassan Moayeri, Karim Rahimi, Mohammad‐Nazir Menbari, Zakaria Vahabzadeh, Mohammad‐Saied Hakhamaneshi, Bijan Nouri, Bayazid Ghaderi, Mohammad Abdi

**Affiliations:** ^1^ Student Research Committee Kurdistan University of Medical Sciences Sanandaj Iran; ^2^ Department of Surgery Faculty of Medicine Kurdistan University of Medical Sciences Sanandaj Iran; ^3^ Department of Molecular Biology and Genetics Gene Expression and Gene Medicine Aarhus University Aarhus Denmark; ^4^ Interdisciplinary Nanoscience Center Aarhus University Aarhus Denmark; ^5^ Cellular and Molecular Research Center Research Institute for Health Development Kurdistan University of Medical Sciences Sanandaj Iran; ^6^ Department of Clinical Biochemistry Faculty of Medicine Kurdistan University of Medical Sciences Sanandaj Iran; ^7^ Department of Epidemiology and Biostatistics Faculty of Medicine Kurdistan University of Medical Sciences Sanandaj Iran; ^8^ Cancer and Immunology Research Center Research Institute for Health Development Kurdistan University of Medical Sciences Sanandaj Iran

**Keywords:** breast cancer, cancer biomarker, Circ‐ELP3, Circ‐FAF1, diagnostic efficiency

## Abstract

**Background:**

Recently, measurement of serum circular RNAs (circRNAs) as a non‐invasive tumor marker has been considered more. We designed the present study to investigate the diagnostic efficiency of serum Circ‐ELP3 and Circ‐FAF1, separately and simultaneously, for diagnosis of patients with breast cancer.

**Methods:**

Seventy‐eight female patients diagnosed as primary breast cancer participated in this study. We measured the level of circRNAs in serum specimens of the studied subjects. A receiver operating characteristic (ROC) curve was plotted and the diagnostic efficiency for both circRNAs was determined.

**Results:**

Compared to non‐cancerous controls, Circ‐ELP3 was upregulated in breast cancer patients (*p‐value* = 0.004). On the other hand, serum Circ‐FAF1 was seen to be decreased in breast cancer patients than controls (*p*‐*value* = 0.001). According to ROC curve results, the area under the curve (AUC) for Circ‐ELP3 and Circ‐FAF1 was 0.733 and 0.787, respectively. Furthermore, the calculated sensitivity and specificity for Circ‐ELP3 and Circ‐FAF1 were 65, 64% and 77, 74%, respectively. Merging both circRNAs increased the diagnostic efficiency, with a better AUC, sensitivity and specificity values of 0.891, 96 and 62%, respectively.

**Conclusion:**

Briefly, our results revealed the high diagnostic value for combined circRNAs panel, including Circ‐ELP3 and Circ‐FAF1 as a non‐invasive marker, in detection of breast carcinomas.

## Graphical abstract

Circ‐ELP3 is overexpressed in breast cancer serum specimens. Circ‐FAF1 is decreased in serum sample of patients with breast cancer. Diagnostic efficiency of serum Circ‐FAF1 is higher than Circ‐ELP3 and both of them have the appropriate diagnostic value for breast cancer detection. Combined measurement of Circ‐ELP3 and Circ‐FAF1 has a high diagnostic value and may be considered a diagnostic biomarker for breast cancer detection.

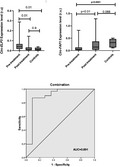

## 1 INTRODUCTION

Breast cancer is the most frequent cancer diagnosed worldwide according to statistics released by the International Agency for Research on Cancer (IARC) in December 2020.^1^ It is expected that there will be 281,550 new cases and 43,600 deaths due to breast cancer within the United States in 2021.^2^ An early diagnosis of breast cancer leads to a successful treatment and, therefore, a great chance of survival. Currently, imaging techniques and measurement of serum tumor markers are utilized for screening of breast cancer patients, although both of them have several limitation including high cost and low diagnostic value.^3^ Although the sensitivity reported for mammography is between 54% and 77%, this technique is the main tool for breast cancer screening.^3^ Histopathological assessment of breast tissue is the gold standard method to confirm the existence of cancer.^4^

Besides, several laboratory markers have been approved to be used in breast cancer screening and monitoring, including cancer antigen 15‐3 (CA 15‐3), carcinoembryonic antigen (CEA) and tissue polypeptide‐specific antigen (TPS). However, they are not reliable enough for breast cancer diagnosis, and recent studies showed contradictory results for utilizing these tumor markers.^5^ Therefore, there is more attention about finding circulating biomarkers as a reliable tool for clinical management of breast cancer.^6^ Among them, recent studies have focused more on the application of molecular biomarkers such as non‐coding RNAs (ncRNAs) because of their high specificity and sensitivity. A new subtype of RNAs is circular RNAs, single‐strand RNA molecules with less than 100 to more than 4,000 nucleotides^7^ and a covalently closed‐loop structure. These molecules are produced through a backsplicing mechanism, in which the downstream 5′‐end of the splice donor joins the upstream 3′‐end splice acceptor and forms a product with a circular structure.^8^, ^9^ In a new classification approach, circRNAs can be divided into two categories including coding and non‐coding circRNAs. Accordingly, coding circRNAs have several elements such as internal ribosome entry site (IRES), an open reading frame (ORF) and specific m6A site which let them to be translated to mRNAs.^10^ Previous studies showed that CircRNAs could involve in various aspects of tumorigenesis like metastasis, invasion, and tumor growth^11^, ^12^, ^13^ and, thus, may be considered as a reliable prognostic and diagnostic marker.

It has been shown that circRNAs are more stable to RNase activity compared to linear form.^14^, ^15^ Besides, due to their long‐time durability in serum, high expression and specificity, circRNAs are considered as favorable biomarkers for diagnosis of various diseases.^16^ Previous studies clearly showed that several circRNAs might act as oncogenes in cancer development such as hsa_circ_0001982^17^ or circGFRA1.^18^ More interestingly, there are some studies that considered circRNAs as a biomarker for cancer management including hsa_circ_0001785^19^ and hsa_circ_100219.^20^ Experimental analysis on hsa_circ_0001785 and hsa_circ_100219 showed a significant alteration in breast tumors for these molecules and, therefore, introduced them as a possible target for treatment or diagnosis of breast cancer.^19^

The gene that encodes hsa_circ_0001785 is elongator complex protein 3 or *ELP3*, a subunit of the acetyltransferase elongator enzyme complex, which is an associated factor with the RNA polymerase II.^21^ Previous studies indicated a significant elevation in ELP3 expression in breast tumors. It has been suggested that ELP3 could enhance breast cancer metastasis via its role on the wobble uridine (U34) of tRNA modification.^22^

FAF1 protein is a potent inhibitor of the TGF‐β signaling pathway. FAF1 overexpression can reduce the metastasis and invasion of breast tumors; thereby, downregulation of FAF1 has a close correlation with increased metastasis in breast cancer.^23^ It was found that hsa_circ_100219 produces from *FAF1* and high level of this circRNA in breast cancer patients can remarkably suppress the proliferation, cell migration, and invasion of cancer cells. Also, hsa_circ_100219, through acting as a miR‐942 sponge, can upregulate the expression of suppressor of cytokine signaling 3 (SOCS3).^24^

Despite the established role of hsa_circ_100219 and hsa_circ_0001785 in breast cancer development, the possible use of these two circRNAs in the clinic is still unclear. Therefore, the present study was designed to investigate the diagnostic value of hsa_circ_0001785 (Circ‐ELP3) and hsa_circ_100219 (Circ‐FAF1) in serum samples of breast cancer patients before and after an intervention to find out whether these circRNAs can utilize as a diagnostic and prognostic biomarker for human breast cancer assessment.

## 2 MATERIAL AND METHODS

### 2.1 Subjects and specimen collection

In this case–control study, we enrolled 78 female patients with breast cancer from Tohid and Kowsar hospitals, Sanandaj, Iran, between June 2019 and February 2020. The inclusion criteria were as follows: (1) histopathological diagnosis of breast cancer; (2) negative history for other types of cancers; (3) negative history for HIV; and (4) having an age more than 18 years old. The diagnosis of breast cancer was performed through immunohistochemical assessment of breast tissue samples by an expert pathologist. All subjects had a negative history of any therapeutic interventions before the first specimen collection. We also enrolled 20 age‐matched control subjects to our study from women who referred to the hospitals for benign breast problems and undergone a mammography procedure. Cancer was ruled out in control subjects through precise examination for the absence of suspected lesions and afterward approved by imaging approaches. This study was approved by the Regional Committee of Ethics of the Kurdistan University of Medical Sciences. For staging and grading the patients, Scarf–Bloom–Richardson criteria and TNM staging system were applied.^4^, ^25^ All clinical, laboratory and pathological details were obtained from patients' medical records.

### 2.2 Sample collection

For specimen collection, 5 ml whole blood was collected from patients before any therapeutic intervention. Six months after beginning treatment (mastectomy, chemotherapy, radiotherapy, or a combination of all), another whole blood sample was obtained from patients. At the same time, a single blood sample was obtained from non‐cancerous subjects. For serum separation, centrifugation was performed at 3500 rpm for 5 min. Subsequently, each separated serum was aliquoted in two vials and was stored at −80°C upon the analysis.^26^, ^27^, ^28^

### 2.3 Quantitative real‐time PCR analysis

According to the manufacturer's protocol, total RNA isolation from serum samples was performed using a Sansure Mag kit (Sansure Biotech, China). Eventually, the quality and quantity of isolated RNA were validated photometrically by a Synergy HTX Multi‐Mode Microplate Reader (BioTek Instruments, Winooski, Vermont, USA). Furthermore, the total RNA integrity was assessed through electrophoretic approach. We synthesized complementary DNA (cDNA) using a PCR Biosystems cDNA synthesis kit (PCR Biosystems, Wayne, Pennsylvania, USA). After that, the real‐time PCR procedure was performed to determine circRNAs expression levels using EvaGreen qPCR Mix Plus (Solis BioDyne, Teaduspargi, Tartu, Estonia) on rotor gene 6000 thermal cycler apparatus (Corbett life science). The primer sequences applied in this study were as follows: circ‐ELP3 forward, 5′‐CAGCATCAGGGATTTGGCAT‐3′, circ‐ELP3 reverse, 5′‐CGACACTGTATTCCGAGGTCTT‐3′, circ‐FAF1 forward, 5′‐ACAAGTATCCCCGTTCGCC‐3′, circ‐FAF1 reverse, and 5′‐CTTCCACATCTCCCGTCTTCC‐3′. Finally, *β‐Actin* gene was used as the reference gene. The relative expression levels were normalized with the *β‐Actin* gene expression. We performed analysis using the comparative cycle threshold 2^−ΔΔCt^ method for RNA expression levels.

### 2.4 Statistical analysis

Data analyzing performed by SPSS 16 (SPSS Inc., Chicago, IL, USA) and GraphPad Prism 8.2.1 (GraphPad Prism Inc., San Diego, CA, USA). We used mean ± standard deviation (SD) for representing the results. Then, for data comparison between the mean of the studied subjects, Mann–Whitney test and one‐way ANOVA analysis were performed, and *p*‐values <0.05 were considered as statistically significant values. Using a receiver operating characteristic (ROC) curve, cutoff values were determined and then the sensitivity and specificity for each circRNAs were calculated.

## 3 RESULTS

### 3.1 Clinical characteristics of study subjects

The mean ages for patients and control group were 46.42 ± 10.94 and 43.21 ± 6.71, respectively (*p‐value* = 0.28). Among cases, 63.6% were positive for human epidermal growth factor receptor‐2 (HER2), and 36.4% were negative. Our results showed that among studied subjects, 71.9% were positive for estrogen receptor (ER) while 28.1% were ER negative. Also, we found that 32.8% of patients were negative for progesterone receptor (PR), and 67.2% were PR positive. Furthermore, 92.9% of patients had invasive ductal carcinoma (IDC), 2.4% had invasive lobular carcinoma (ILC), and 4.8% had ductal carcinoma in situ (DCIS). Histological grading and staging were performed through the pathological assessments for all patients. Among them, 25.6% had stage 0–I, 34.9% stage II, 27.9% with stage III, and 11.6% had stage IV. The frequency of clinical grading 1, 2, and 3 in patients was 18.6%, 55.8%, and 25.6%, respectively. Patients in this study underwent three different treatment approaches, 35.9% chemotherapy, 14.1% surgery, and the remaining 50% experienced a combination of the two medical interventions. All demographic and clinical data are shown in Table 1.

**TABLE 1:** Clinical characteristics of the studied subjects (*n* = 78)

Characteristics	% of subjects
Age (years)	
≤43	31.8
>43	68.2
ER	
Positive	71.9
Negative	28.1
PR	
Positive	67.2
Negative	32.8
HER2	
Positive	63.6
Negative	36.4
Tumor stage	
0–I	25.6
II–III	62.8
IV	11.6
Tumor grade	
1	18.6
2	55.8
3	25.6
Treatment	
Surgery	35.9
Chemotherapy	14.1
Combined	50.0

Abbreviations: ER, estrogen receptor; HER2, human epidermal growth factor receptor‐2; PR, progestin receptor.

### 3.2 Expression levels of the studied circRNAs

**FIGURE 1:**
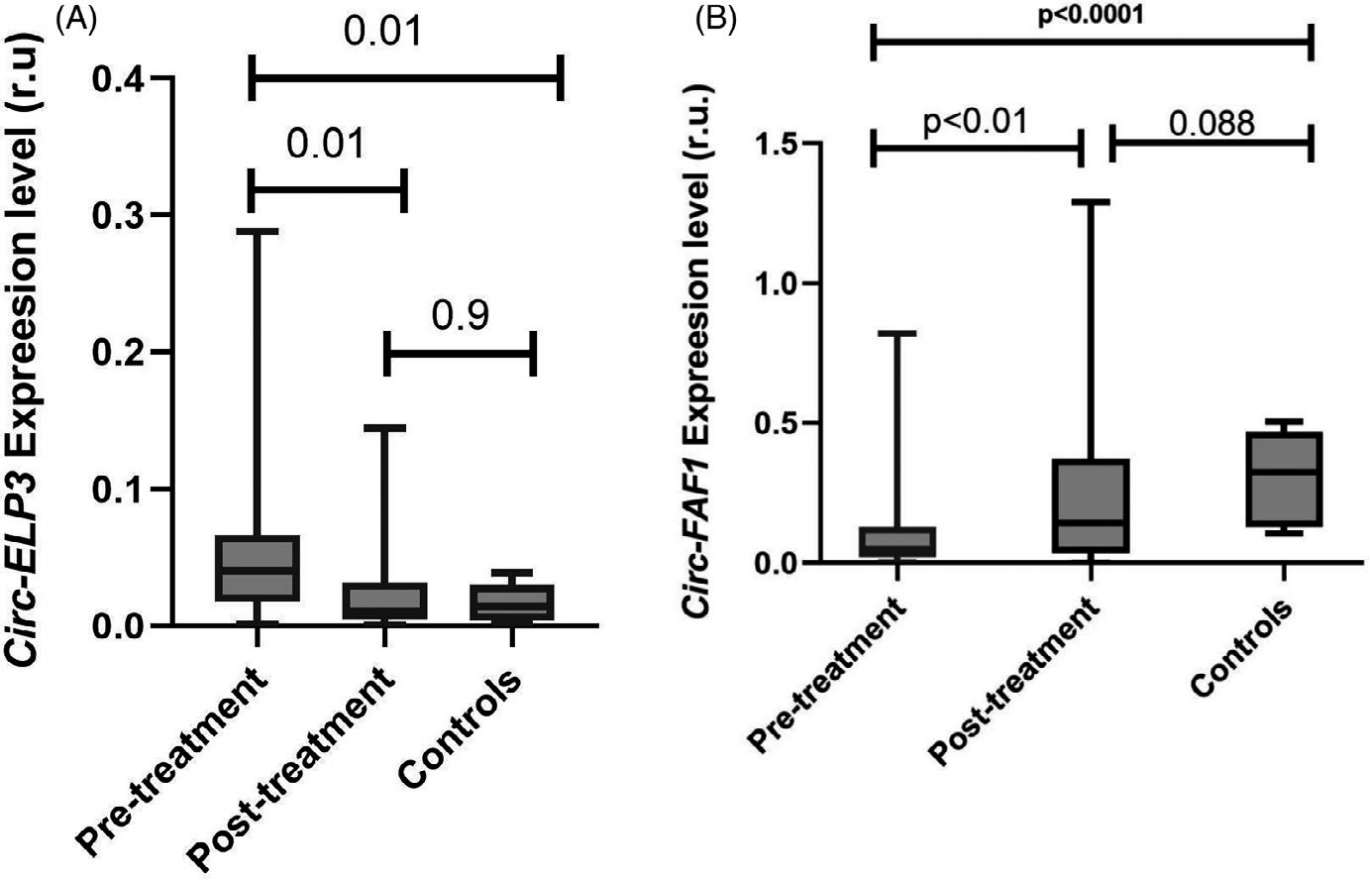
Expresion level of Circ‐ELP3 in studied groups: (A) Serum Circ‐ELP3 was higher in patients compared to controls and tcaused this circRNA to be decreased. (B) In contrast to Circ‐ELP3, Circ‐FAF1 was downregulated in new cases of breast cancer patients and after treatment it was seen as an increase in serum level of this circRNA

The expression level of two studied circRNAs hsa_circ_0001785 (Circ‐ELP3) and hsa_circ_100219 (Circ‐FAF1) is illustrated in Figure 1. Our results showed that the circulating level of hsa_circ_0001785 (Circ‐ELP3) in breast cancer patients before treatment was upregulated compared with controls (*p‐value* = 0.0106), while after treatment, the level of this circRNA was significantly decreased compared to pre‐treatment status (*p‐value* = 0.01) and, moreover, this value had no statistically significant difference with control group (*p‐value* = 0.9451) (Figure 1A). As illustrated in Figure 1B, the circulating level of hsa_circ_100219 (Circ‐FAF1) in serum specimen of patients before treatment was significantly lower than controls (*p* < 0.0001), while the expression level showed a statistically significant overexpression after treatment (*p‐value* = 0.0069). Additionally, we evaluated the relation between clinical characteristics of studied subjects with the expression level of Circ‐ELP3 and Circ‐FAF1. There was no statistically significant difference between the expression level of circRNAs, hsa_circ_0001785 (Circ‐ELP3) or hsa_circ_100219 (Circ‐FAF1), in pre‐treatment status with patients' age, breast cancer clinical stage and grade, and the affected breast side (Tables 2 and 3). The correlation between circRNAs levels and the other clinical characteristics including receptors (HER2, ER, PR, and Ki67) and the treatment efficiency are summarized in Tables 2 and 3.

**TABLE 2:** Relationship between Circ‐ELP3 and clinical features in patients

Variable	Subclass	Mean ± SD	*p*‐value
Age	≤43	0.0973 ± 0.097	0.1247
	>43	0.047 ± 0.47	
Breast cancer type	IDC	0.063 ± 0.074	0.9139
	ILC	0.036 ± 0.0	
	DCIS	0.046 ± 0.0	
Histological grade	1	0.074 ± 0.083	0.063
	2	0.046 ± 0.031	
	3	0.060 ± 0.083	
Clinical stages	0–I	0.075 ± 0.083	0.1443
	II–III	0.054 ± 0.062	
	IV	0.028 ± 0.029	
ER status	Positive	0.053 ± 0.057	0.40
	Negative	0.073 ± 0.086	
PR status	Positive	0.054 ± 0.061	0.36
	Negative	0.070 ± 0.081	
HER2 status	Positive	0.029 ± 0.023	0.0951
	Negative	0.072 ± 0.079	
Breast side affected	Right	0.057 ± 0.064	0.7871
	Left	0.049 ± 0.60	

**TABLE 3:** Relationship between Circ‐FAF1 and clinical features in patients

Variable	Subclass	Mean ± SD	*p*‐value
Age	≤43	0.16 ± 0.23	0.70
	>43	0.081 ± 0.89	
Breast cancer type	IDC	0.106 ± 0.15	0.86
	ILC	0.146 ± 0.0	
	DCIS	0.033 ± 0.0	
Histological grade	1	0.1122 ± 0.1735	0.96
	2	0.097 ± 0.13	
	3	0.096 ± 0.025	
Clinical stages	0–I	0.1083 ± 0.1779	0.1410
	II–III	0.107 ± 0.108	
	IV	0.08594 ± 0.086	
ER status	Positive	0.122 ± 0.17	0.91
	Negative	0.085 ± 0.1	
PR status	Positive	0.11 ± 0.17	0.67
	Negative	0.10 ± 0.13	
HER2 status	Positive	0.1166 ± 0.13	0.74
	Negative	0.1022 ± 0.15	
Breast side affected	Right	0.12 ± 0.091	0.08
	Left	0.054 ± 0.076	

### 3.3 Diagnostic value of studied circRNAs

**FIGURE 2:**
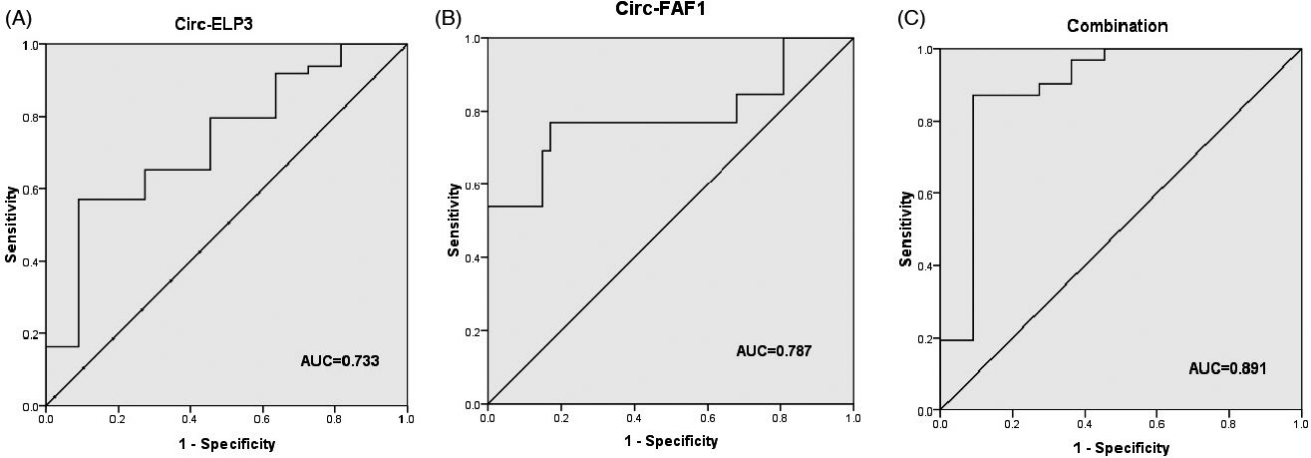
ROC curve analysis of Circ‐ELP3, Circ‐FAF1, and combination of the two circRNAs: (A) ROC curve analysis results of Circ‐ELP3. (B) ROC curve analysis results of Circ‐FAF1. (C) The combination of Circ‐ELP3 and Circ‐FAF1

By drawing a ROC curve, we determined the diagnostic values of hsa_circ_0001785 (Circ‐ELP3) and hsa_circ_100219 (Circ‐FAF1) for diagnosis of breast cancer (Figure 2A,B). The cutoff value and the area under the curve (AUC) for hsa_circ_0001785 (Circ‐ELP3) were 0.028 (r.u.) and 0.733 (95% confidence interval (CI) 0.573–0.892), respectively. We also determined the cutoff point of hsa_circ_100219 (Circ‐FAF1) as a biomarker for breast cancer. The corresponding value for this circRNA was 0.064 (r.u.), and the AUC was 0.787 (95% CI 0.613–0.962). Then, we used the above cutoff values to calculate sensitivity and specificity of hsa_circ_0001785 (Circ‐ELP3) and hsa_circ_100219 (Circ‐FAF1). The results are shown in Table 4; as mentioned in this table, Circ‐FAF1 has higher diagnostic efficiency for breast cancer detection according to the AUC value.

**TABLE 4:** Diagnostic value of Circ‐ELP3 and Circ‐FAF1 in separate and combined form for breast cancer detection

	AUC	Sensitivity (%)	Specificity (%)
Circ‐ELP3	0.733	65	64
Circ‐FAF1	0.787	77	74
Circ‐ELP3 + Circ‐FAF1	0.891	96	62

Finally, we combined the two circRNAs to see whether it produce a better diagnostic value for detection of breast cancer. As it is shown in Figure 2C, the combination of them showed higher AUC. Furthermore, using a combined panel, the sensitivity of the test was highly increased and showed higher diagnostic efficiency for breast cancer patients compared to a single test panel (Table 4).

## 4 DISCUSSION

Successful treatment of breast cancer patients completely relies on its diagnosis in early stages. Imaging techniques including mammography, magnetic resonance imaging (MRI), positron‐emission tomography (PET), computed tomography (CT), and single‐photon emission computed tomography (SPECT) along with laboratory assessment of biochemical tumor markers are two important diagnostic tools for determination of patients with breast cancer. A great number of biochemical markers have been shown that could be utilized for breast cancer diagnosis including proteins and DNA.^29^ Among circulating tumor markers, measuring serum level of cancer antigen 15‐3 (CA15‐3) or carcinoembryonic antigen (CEA) is more recommended. The diagnostic value for CEA and CA15‐3 has been studied well. In a recent study by Uygur et al.,^30^ measuring serum CEA and CA 15‐3 had shown highest sensitivity for hormone receptor and highest specificity for HER2 status (88.17% and 60%, respectively). In another study by Wand et al.,^31^ CEA and CA15‐3 showed low sensitivity (56.7% and 44.5%, respectively) and high specificity (92% and 84.5%, respectively) for diagnosis of metastatic breast cancers. A recent meta‐analysis about the diagnostic efficacy of CEA and CA15‐3 in patients with breast cancer revealed that higher plasma CEA and CA15‐3 are correlated with poor disease‐free survival and overall survival, and therefore, suggested that they might be evaluated anytime if possible.^32^ Collectively, utilizing these tumor markers in breast cancer diagnosis is still controversial. Besides, various imaging methods have several limitations such as high cost and low sensitivity or specificity.^29^ Therefore, more studies have focused on introducing novel circulating tumor markers for breast cancer diagnosis and monitoring of treatment.

The aberrant expression of circRNAs in breast cancer has already been shown in previous studies. In a recent study by Zhang and colleagues,^33^ the expression level of circular RNA La‐related RNA‐binding protein 4 (circ‐LARP4) and its impact on tumor characteristics, prognosis, and treatment of breast cancer were evaluated in clinical tissue samples and cell culture. They showed a significant decline in circ‐LARP4 level in breast cancer tissues. Besides, the expression of this circRNA was associated with tumor size, TNM stage, disease‐free survival, and overall survival. They also showed that downregulation of circ‐LARP4 could stimulate breast cancer progression. In another study by Li et al.,^34^ the possible relation between circular RNA VRK serine/threonine kinase 1 (circ‐VRK1) with clinical outcomes in breast cancer patients and characteristics of cancer cells were studied. They found that this circRNA is downregulated in breast cancer tissues and the expression level of circ‐VRK1 was associated with tumor size and TNM stage, and could be considered as an independent predictor of better overall survival. Similar result was shown in breast cancer cell line and, more importantly, upregulating circ‐VRK1 suppressed cell proliferation and activated cell apoptosis in studied cell lines. Lu et al.^20^ investigated 1155 circRNAs in breast cancer tissue, among them, 715 circRNAs were upregulated and 440 showed downregulation. According to the results, circ_103110, hsa_circ_104689, and hsa_circ_104821 levels were overexpressed, and hsa_circ_006054, hsa_circ_100219, and hsa_circ_406697 were downregulated, and hsa_circ_100219 showed the maximum diagnostic value. Hu et al. investigated circRNAs expression profile in breast cancer and non‐cancerous tissues and revealed that 54 circRNAs were upregulated and 94 downregulated. Among them, they found that hsa_circ_0008673 upregulated in breast tissues and had the highest diagnostic value in plasma specimens. The calculated diagnostic values including AUC, cutoff, specificity, and sensitivity for hsa_circ_0008673 were 0.833, 1.380, 97.1%, and 55.0%, respectively, which shows higher specificity and lower sensitivity as compared to our results. Also, they showed that there is a direct association between has‐circ‐0008673 and several clinical indices including larger tumor size, distant metastasis, positive estrogen receptor (ER) status, positive progesterone receptor (PR) status, and might use as a prognostic predicator of overall survival (OS) and disease‐specific survival (DSS).^35^ In another study by Yin et al.,^19^ expression profile of 41 circRNAs with an aberrant expression was assessed, and demonstrated 19 circRNAs with an increased expression and also 22 downregulated circRNAs. They found that hsa_circ_0001785 (Circ‐ELP3) has a high diagnostic value for detecting breast cancer. Subsequently, statistical analysis performed in this study revealed that hsa_circ_0001785 (Circ‐ELP3) has an acceptable diagnostic value (AUC = 0.715, 95% CI = 0.825, 0.595–1.000) as compared to CEA and CA 15‐3 and, therefore, could be considered as a potential biomarker for detecting breast cancer. With regard to hsa_circ_100219 (Circ‐FAF1), our results revealed an AUC of 0.787 (95% CI 0.613–0.962), which showed an acceptable diagnostic efficiency for breast cancer detection. In line with previous studies, we showed that the serum level of hsa_circ_0001785 (Circ‐ELP3) in new cases of breast cancer is higher than control subjects. More interestingly, Circ‐ELP3 downregulated in patients underwent medical interventions including surgery and/or chemotherapy. The underlying hypothesis for this decrease could be explained by the effect of therapeutic procedure on tumor size and, therefore, decline in Circ‐ELP3 expression and excretion from tumor cells. On the other hand, Circ‐FAF1 showed a significant lower serum level in patients compared to the controls. This result was in accordance with previous studies. Lu et al.^20^ confirmed that the hsa_circ_100219 (Circ‐FAF1) level declines in breast cancer tissue and leads to initiation or facilitating cell apoptosis. To the best of our knowledge, our study for the first time has investigated the possible use of Circ‐ELP3 and Circ‐FAF1 as a combined double marker for detection of breast cancer. The diagnostic efficiency for Circ‐FAF1 was slightly higher than Circ‐ELP3 as according to the AUC, sensitivity, and specificity. These values were the highest ones that have ever been reported for this circRNA. Yin W et al.^19^ studied the diagnostic value for Circ‐ELP3 and showed that the AUC, specificity, and sensitivity for this circRNA were 0.784, 75.6%, and 78.6%, respectively. Lu et al.^20^ reported the AUC, specificity, and sensitivity values for circ‐FAF1 as 0.78, 71%, and 69%, respectively. The determined sensitivity and specificity for hsa_circ_0001785 (Circ‐ELP3) in our study was lower than previous studies. In contrast to the other reported values for circRNAs, we found a high diagnostic efficiency for hsa_circ_100219 (Circ‐FAF1) in the present study. More interestingly, the results for combined panel showed even better diagnostic efficiency for breast cancer detection and proved that this panel could be considered as a potential marker for breast cancer management.

In conclusion, our results revealed an upregulation in Circ‐ELP3 and, in contrast, a downregulation in Circ‐FAF1 in serum specimens of patients with breast cancer while the levels of these circRNAs showed a decrease and an increase values after treatment, respectively. Furthermore, because of high diagnostic efficiency, Circ‐ELP3 and Circ‐FAF1 could be considered as a potential biomarker for breast cancer detection, especially when used in combination.

## CONFLICT OF INTEREST

Mr. R Omid‐Shafa'at declares no potential conflicts of interest with respect to the research, authorship, and/or publication of this article. Dr H Moaieri declares that he has no conflict of interest. Dr K Rahimi declares that he has no conflict of interest. Dr MN Menbari declares that he has no conflict of interest. Dr Z Vahabzadeh declares that he has no conflict of interest. Dr MS Hakhamaneshi declares that he has no conflict of interest. Dr B Nouri declares that he has no conflict of interest. Dr B Ghaderi declares that he has no conflict of interest. Dr M Abdi has received research grants from Kurdistan University of medical sciences.

## AUTHOR CONTRIBUTIONS

R.O‐Sh. carried out the experiments. M‐N.M. and M‐S.H. helped and supervised the project. B. Gh, Z.V., B.N., and H.M. conceived and planned the experiments. M.A. took the lead in project, conceived the original idea, supervised the project, analyzed the results, and wrote the manuscript.

## INFORMED CONSENT

Informed consent was obtained from all individual participants included in the study.

## Data Availability

The data that support the findings of this study are available from the corresponding author upon reasonable request.
